# Supplementary material for: Data-driven CAD-CAM vs traditional total contact custom insoles: A novel quantitative-statistical framework for the evaluation of insoles offloading performance in diabetic foot
Source: PLoS One. 2021 Mar 4;16(3):e0247915. doi: 10.1371/journal.pone.0247915 (PMC7932531; doi:10.1371/journal.pone.0247915)
Supplement: Attachment — Submitted filename: Response to Reviewers PONE-D-20-14175_final review.docx [file pone.0247915.s002.docx]

PONE-D-20-14175
Data-driven CAD-CAM vs traditional total contact custom insoles: a novel quantitative-statistical framework for the evaluation of insoles offloading performance in diabetic foot
PLOS ONE

Dear Author(s)

Thank you for submitting your manuscript to PLOS ONE. After careful consideration, we feel that it has merit but does not fully meet PLOS ONE’s publication criteria as it currently stands. Therefore, we invite you to submit a revised version of the manuscript that addresses the points raised during the review process.

Although the reviewers impressions have been quite positive, they do address some major components requiring changes. Please respond to their comments in detail.

Please submit your revised manuscript by Aug 31 2020 11:59PM. If you will need more time than this to complete your revisions, please reply to this message or contact the journal office at [plosone@plos.org](mailto:plosone@plos.org). When you’re ready to submit your revision, log on to <https://www.editorialmanager.com/pone/> and select the ‘Submissions Needing Revision’ folder to locate your manuscript file.

Please include the following items when submitting your revised manuscript:

- A rebuttal letter that responds to each point raised by the academic editor and reviewer(s). You should upload this letter as a separate file labeled ‘Response to Reviewers’.
- A marked-up copy of your manuscript that highlights changes made to the original version. You should upload this as a separate file labeled ‘Revised Manuscript with Track Changes’.
- An unmarked version of your revised paper without tracked changes. You should upload this as a separate file labeled ‘Manuscript’.

If you would like to make changes to your financial disclosure, please include your updated statement in your cover letter. Guidelines for resubmitting your figure files are available below the reviewer comments at the end of this letter.

If applicable, we recommend that you deposit your laboratory protocols in [protocols.io](http://protocols.io/) to enhance the reproducibility of your results. Protocols.io assigns your protocol its own identifier (DOI) so that it can be cited independently in the future. For instructions see: <http://journals.plos.org/plosone/s/submission-guidelines#loc-laboratory-protocols>

We look forward to receiving your revised manuscript.

Kind regards,

Kevin Mattheus Moerman, Ph.D.
Academic Editor
PLOS ONE

Journal Requirements:

When submitting your revision, we need you to address these additional requirements.

Please use the space provided to explain your answers to the questions above. You may also include additional comments for the author, including concerns about dual publication, research ethics, or publication ethics. (Please upload your review as an attachment if it exceeds 20,000 characters)

**ANSWERS TO REVIEWERS**

**Please find hereinafter our detailed answer** **to each point raised by the academic editor and reviewer(s)**

**PREMISE**

We want to thank the reviewers for their suggestions, comments and critics that resulted helpful and useful to stimulate us to improve the submitted paper.

We were working hard to rewrite essential paragraphs and sections to fulfil their suggestions and to explicit all the underlying concepts we left as “tacit” and “implicit” in the previous version.

Indeed, after receiving comments and suggestions from the reviewers, we realised that the main reason that led us to develop the proposed quantitative statistical framework, i.e. the necessity to approach and manage the “Gait natural variability”, was not given as an explicit motivation. For such a reason we modified the manuscript in the Introduction, Methods, Results, and Discussion sections to introduce and discuss at length such a concept.

We do believe that now the final revision of the paper is more understandable and more comprehensive. Each point raised by the academic editor and reviewer(s) has been considered in the revision.

In the following, we provide a specific answer to all the raised points:

**N.B. Please note all the given line numbers in the following are referred to the unmarked version of the manuscript.**

Reviewer #1: Thank you for your publication and contribution to an area that is lacking robust evidence.
Overall, I am struggling to understand the quantitative statistical framework -this needs to be more explicit within your article. What is it, what was the method, make it more explicit in the results section.

We thank the reviewer #1 for such a comment.

We realised that the main reason that led us to create the quantitative statistical framework (QSF), i.e. the creation of a method to manage the natural gait variability appropriately was not openly declared. So, we modified the Introduction section where we raised such an issue explaining why such an issue has to be approached.

Please refer to lines 104-114

Then we added in the Methods section a full paragraph to clearly describe what the QSF is, its meaning, and all the requirements to properly manage data analysis.

Please refer to lines 340-350

In the Results section, the importance of QSF is straightforward.

Please refer to lines 353-444

In the Discussion section, we discussed at length how the proposed QSF is the adequate necessary tools to handle the quantitative underfoot pressure measurements, considering the intrinsic gait variability, showing its superiority to the direct 200kPa threshold.

Please refer to lines 466-493

line 27 throughout the article you interchange use the terms insoles and orthotics and sometimes add in footwear - can you be consistent and use one term throughout (eg line 29)

We thank the reviewer #1 for such a comment.

We recognised that this could be an issue. To be as much general as possible, because several healthcare disciplines may be involved in the provision of footwear and insoles for people with diabetes, we referred to the definitions proposed in van Netten et al., where the terms “custom-made insoles” and “custom-made in-shoe orthosis/orthotic” are equivalent and in a such a way they will be used throughout this paper.

Please refer to lines 69-74

line 50 - neuropathy and neuropathic in same sentence

Removed

line 55 add in ‘the’ before foot

Done

line 55 very long sentence - split into 2

Done

line 60 add a full stop

Done

line 61 footwear is also an important aspect of reducing foot pressures and usually works in conjunction with the insole....especially as you refer to outer sole in line 74

Added in the Introduction a clarification of the footwear role

Please refer to lines 65-68

We also added in the Discussion section further considerations about the footwear role and the difficulty in differentiating the effect of the different insole and footwear features in offloading the neuropathic diabetic foot as found in the very recent review published by reviewer#1. We found this recent review very useful to strengthen some of the concepts we wanted to stress in our paper.

Please refer to lines 507-521 and 568-573

ln 85 add ‘an’ at start of sentence

Sentence removed

ln 85/86 - this sentence does not make sense

Sentence removed

ln 87 remove also

Done

ln 88 clarify ‘periodic systematic control’

Added in the Introduction a clarification.

Please refer to lines 97-99

ln 90 remove ‘still’

Done

ln 91 remove nevertheless

Done

ln 95 remove up

Done

ln 99 write info in full

Done

Ln 109 - was acute vascular problems an exclusion?

Thank you for noticing.

YES. We added in the exclusion criteria

Please refer to line 136

ln 121 - peripheral neuropathy consists of motor, sensory and autonomic - do you mean sensory in this sentence?

Added sensory

ln 133 - who was double blinded?

A clarification was added in the Methods.

Please refer to lines 159-161

ln 133 clarify subsequent

Removed

ln 134 special sandals requires better description and company

A clarification was added in the Methods.

Please refer to lines 155-156

ln 135 neutral flat insole - why do you need to use neutral? - flat insole would suffice but requires more description eg materials, pictures

The word neutral was removed throughout the paper

A clarification was added in the Methods section for flat insole material and manufacturer.

Please refer to lines 169-172

ln 136 - how much time between 1st and 2nd sessions?

A clarification was added in the Methods.

Please refer to lines 213-217

were the sandals worn just for the testing or also between sessions by participants?

A clarification was added in the Methods.

Please refer to line 217

ln 137-141 - rewrite to make more readable

Rewritten in the Methods.

Please refer to lines 161-167

ln 143 - clarify all foot othoses are TCCI and cad cam (flat insole is also a device)

Please see above

ln 143 and ln 151 contradict as you say eva used only and then you add a top cover

Rewritten in the Methods.

Please refer to lines 169-171

ln 169 - clarify if TCCI and Cad cam orthoses; incidentally who made the flat insoles?

Clarified.

Please refer to line 197

For flat insole manufacturer, please read above

ln 182- reword for clarification (eg each participant recieved ...)

Rewritten in the Methods.

Please refer to lines 213-227

ln 186 - clarify if experimenatl session 1 or 2

Rewritten in the Methods.

Please refer to lines 213-227

ln 186 clarify if the two testing conditions are TCCI and cad cam

Rewritten in the Methods.

Please refer to lines 213-227

ln 206 experimental sandals

Added the word “experimental”.

Please refer to line 244

ln 225 - how did the speed of testing session 1 compare with session 2?

A clarification was added in the “In-Shoe Dynamic Plantar Pressure Measurement” sub-section.

Please refer to lines 245-252

ln 238 - clarify why not necessary to use masking to reader

Rewritten in the Data Analysis.

Please refer to lines 287-290

the parametric and non-parametric analysis - please clarify the purpose of these tests (eg we used the test to determine....)

Rewritten completely by introducing three specific sub-sections

Population sample size determination

Sample size determination (number of required steps)

Non-Parametric Statistical analysis.

Please refer to lines 300-339

ln 331 - you say descrptive but i cannot find them

The whole sentence was removed (it was a typing error left by chance)

ln 338 - you are not examining the distribution of roi’s - reword

Rewritten in the Results.

Word “distribution” removed.

Please refer to line 402

ln 341 - define beneficial

Rewritten in the Results.

Please refer to lines 403-404

percentage reduction in pressure would be a useful addition to your absolute data as it would account for the large variations in participants barefoot pressure data that absolute values do not show

Added in Table 3

Please refer to lines 431-435

ln 389 - robust? i am not clear how it is robust as you have not presented any justification for this

Reworded as “sound”.

ln 396 better performance - in terms of what?

Rewritten in the Discussion.

Please refer to lines 452-453

ln 399 - valuable point but not appropriate in this paragrapgh

Removed

ln 411 - ‘perfect’ is a very strong word to use in this context

Removed

ln 418 - what tests did you use to justify its reliability? i don’t think you did so can’t claim this

Rewritten in the Discussion the word “reliability” was removed.

Please refer to lines 466-467

ln 447-448 i am still not sure what the QSF framework is

Please see above Introduction, Methods, Results, Discussion.

ln 452 - what about different gait strategies and changes over time?

We explained throughout the paper that QSF method allows precisely to evaluate the influence of the different walking strategies as well as to monitor their changes over time

ln 455 full stop missing

Done

Reviewer #2: The paper presents a comparative study between three different types of orthotics in order to evaluate which is the best for reducing the foot plantar pressure. The evaluation is conducted considering 30 patients and different production technologies.
The manuscript is well structured and clear in its contents. The analysis methodology is presented in detail. The conclusions are supported by well-analysed clinical data.
Custom made CAD-CAM insoles have been designed considering a single CAD tool. For the reviewer, this is the main limitation of the work. The effectiveness of an insole is strictly dependent on its geometry and therefore on the algorithm/software used to define its geometry. The reviewer recommends adding information regarding the offloading algorithm used to generate the 3D geometry of orthotics, as well as the CAD modelling procedure. As a future development, it would be interesting to see a comparison between orthotics developed with different modelling software

We thank the reviewer #2 for her/his comments.

We added information about the CAD modelling procedure that was not disclosed to the authors because of Intellectual Property reasons

Please refer to lines 206-209, where we added:

“The automated design algorithm details are the intellectual property of Guantificio Altotiberino ECOSANIT Calzature Snc, Anghiari, Italy, and they were not disclosed to the authors being not strictly necessary for the present study.”

We also added specific considerations in the limitations of the study paragraph.

Please refer to lines 591-597

Reviewer #3: This is an interesting topic of investigation, as we are in need of effective offloading insoles for the diabetic foot. The authors introduce a new framework to analyse pressure pictures and obtain results from, but I am trying to find the real benefits of this approach over regular in-shoe peak pressure analysis from the pressure distribution pictures, which is quite a straightforward analysis itself. What is the problem we have that stimulates the development of this new framework? How is it going to help us better to interpret in-shoe pressure pictures?

We thank the reviewer #3 for her/his comments.

Please, refer to the above-written PREMISE.

Refer as well to modifications we introduced in the paper.

We explained in various sections (Introduction, Methods, Results, Discussion) the motivation which led us to propose the QSF.

We clarified why QSF provides better solution respect to direct 200kPa threshold methods proposed in the literature

Specific comments

Abstract
- Line 33: this is not a double-blind controlled trial. It is a cross-sectional study design

Removed double-blinded in the abstract and added explanations in the Methods section.

Please refer to lines 159-161

- Line 36: very few quantitative results are reported compared to lots of text in the abstract, which is a bit odd given the QSF, a quantitative method that the authors are using

Added some more numerical results considering the Abstract length limit of 300 words.

Introduction
- In line 63, the authors refer to poor adherence being a barrier to clinical success with insole designs. This is supported by trial data from Bus et al.1, an important reference that is missing from the reference list.

Added in the reference list

- In line 76 the authors state: “Plantar pressure can be redistributed but not eradicated and reducing stress at one location may simply displace the risk of ulceration to a different area of the foot.” This is true for the average pressure obtained, but not necessarily for the peak of the plantar pressure, a parameter that is mostly reported in the diabetic foot literature. I would suggest to specify this in the paper.

We are not sure to understand the following part of the comment from a theoretical point of view

“but not necessarily for the peak of the plantar pressure, a parameter that is mostly reported in the diabetic foot literature. I would suggest to specify this in the paper”

In any case from our results, it has been demonstrated that the mean peak pressure maps were indeed modified by custom-made insoles respect to the flat insole. Potentially a wrong offloading modification, in the custom-made insoles, can displace R-ROIs (i.e. cells clusters that resulted, with ascertained statistical significance, pressure values, higher than 200kPa risk of ulcer) from one specific underfoot region to a different one.

- In line 91 the authors state: Nevertheless, the strategies to design the optimal offloading insoles have to be fully standardised. In the absence of such standardisation, outcomes for digital quantitative data-driven approaches can be debatable in comparison of traditional foot orthoses supply chains [14].” Recently, two papers have been published, specifically addressing this topic, which stress that standardisation and data-drive design protocols are needed, although not necessarily via CADCAM design principles2,3

Added in the reference list

- In line 95, the authors state their aim to: “Firstly, we wanted to build up a robust quantitative-statistical framework (QSF) to analyse in-shoe pressure measurements to propose a way to standardise the evaluation of offloading insoles’ design and subsequent monitoring steps.” But in the section before the aim, the authors state that the design of insoles needs to be standardised. So, is it the evaluation of the insole design or the design itself that has to be standardised, as these are two different things? If referring to the design, this is more valuable than when referring to the evaluation.

We clarified in the Methods and the Discussion, how QSF could help in the process of standardisation of both evaluation and design protocols.

Please refer to lines 340-350 (Evaluation)

Please refer to lines 475-482 and 549-563 (Design)

Methods
- Why were mostly risk class 1 subjects recruited, as the insoles studies would normally apply more to a risk class 2 or 3 patient?

As explained in the Methods, 220 patients were screened to obtain the needed 30 (number of patients determined through statistical inference see Population sample size determination sub-section) patients population satisfying the inclusion/exclusion criteria. Once the necessary number of patients was reached, the research could start.

- Line 171: “Plantar pressure data, which were integrated into its algorithms for the manufacture of the custom insoles, were also provided.” Which plantar pressure data is meant? More detail is required here

Added and clarified in the Methods section

Please refer to lines 199-203

- Line 182: why was a double-blind design considered important for this study? If not blinded, how would that have affected the results?

Added and clarified in the Methods section

Please refer to lines 159-161

- Line 234: Why were “The total surfaces of R-ROIs per each foot“ chosen as the primary variable for comparison between offloading insoles, and why not the peak pressure, which is the most commonly reported parameter. And what was the rationale for using a parameter like R-ROI and not analysing the pressure values in a more straightforward fashion as done by others?

Please, refer to the above-written PREMISE.

Refer as well to modifications we introduced in the paper.

We explained in various sections the motivation which led us to propose the QSF.

We clarified why QSF provides better solution respect to direct 200kPa threshold methods proposed in the literature

The R-ROIs (i.e. cells clusters that resulted, with ascertained statistical significance, pressure values higher than 200kPa risk of ulcer) are implicitly considering the pressure values as explained. Their sizes provide quantitative information on how much the offloading modifications in the custom-made insole resulted successfully.

- Line 241 onwards: the authors present the study, as the title shows as a “a novel quantitative-statistical framework”. It is not clear what is novel about it and what the ‘statistical framework’ is, as it seems that straightforward parametric and non-parametric tests are used, comparing the different conditions in a cross-sectional study fashion.

Please, refer to the above-written PREMISE.

Refer as well to modifications we introduced in the paper.

We explained in various sections the motivation which led us to propose the QSF.

We clarified why QSF provides better solution respect to direct 200kPa threshold methods proposed in the literature

Besides, we added in the Methods section, a full paragraph to clearly describe what the QSF is, its meaning, and all the requirements to properly manage data analysis.

Please refer to lines 340-350

Furthermore, sub-sections in the Methods have been rewritten entirely to explain better the statistical test applied. In particular, three specific sub-sections have been introduced:

Population sample size determination

Sample size determination (number of required steps)

Non-Parametric Statistical analysis.

Please refer to lines 300-339

- Line 257: why is for the power calculation of “underfoot pressure redistribution” the number of steps indicated, whereas normally, and for the primary parameters of R-ROI, the number of patients is reported?

Sub-sections in the Methods have been rewritten entirely to explain better the statistical test applied, In particular, three specific sub-sections have been introduced:

Population sample size determination

Sample size determination (number of required steps)

Non-Parametric Statistical analysis.

Please refer to lines 300-339

Results
The results section is difficult to read and requires improvement

Results section has been fully rewritten

- Line 279 The authors use a subheading, but this is the only one in the results section, so redundant

Removed

- Line 293 to 306 reads as a legend of the Figure and should be moved to the legend, so that the results section only contains the results of the analyses
- The same for lines 319 to 325

Moved into the legends

- Lines 358 – 380 contain a lot of explanation rather than results, that one would expect in the methods or discussion section

Partially moved to the Discussion section and partially rewritten.
Please refer to lines 581-590

- There are 8 figures and 3 tables, which is a lot for a study using just 2 parameters, straightforward analyses and very few quantitative results. In particular, the results on ROI-R are sometimes difficult to follow

Figure 7 and 8 have been removed

- I don’t see any reference in the results to the regions that are most important to offload in the neuropathic diabetic patient, like the hallux, 1st metatarsal head, where foot ulcers most commonly occur. How does the framework take that into account, as some regions are more important to analyse than others? And how does it do that in an efficient way, as from peak pressure distribution pictures from the in-shoe pressure measurement, this information can be directly observed.

Please reread the revised version of the paper. We are confident now after such revision that everything should be clear. In any case, by seeing the Results section, either in Figure 4 or Figure 5, the R-ROIs are precisely indicating that the patients considered in such examples were presenting at-risk areas under the metatarsal heads and halluces.

Discussion

- Line 389 – 405 read as an introduction. I suggest to discuss the results of the study sooner

They were partially removed. Discussion section rewritten; results discussed sooner.

- Line 418. The authors state that “ The quantitative-statistical framework herein introduced demonstrated its reliability and usefulness in identifying high-risk areas with ascertained statistical significance (i.e. R-ROIs), representing a marked improvement compared to the methods based on the direct 200kPa cut-off.” Where in the results can I find results on the reliability and usefulness of the framework and how robust it is, as the authors state in the conclusions? And based on which outcome do they conclude that this is a marked improvement compared to using the 200 kPa cutoff method?

Please reread the revised version of the paper. We are confident now after such revision that everything should be clear.

The word “reliability” has been removed not to generate confusion with “statistical reliability.”
Please refer to line 466

- Line 438: “ Long term monitoring is another reason to prefer the CAD-CAM design approach….”. This is contrary to a recent study that shows that handmade custom-made insoles provide more pressure relief than CADCAM insoles.3

We are not sure to understand the above comment, in that it does not appear in any contrast with the cited paper 3. Moreover, such a study proved that considering additional information, data-driven modifications improve the offloading efficacy on handmade and CAD-CAM design as well.

In any case, we added further considerations, including this latter in the Discussion section.

Please refer to lines 524-534

References

1. Bus SA, Waaijman R, Arts M, et al. Effect of custom-made footwear on foot ulcer recurrence in diabetes: a multicenter randomised controlled trial. Diabetes Care 2013; 36(12): 4109-16.
2. Bus SA, Zwaferink JB, Dahmen R, Busch-Westbroek T. State of the art design protocol for custom made footwear for people with diabetes and peripheral neuropathy. Diabetes Metab Res Rev 2020; 36 Suppl 1: e3237.
3. Zwaferink JBJ, Custers W, Paardekooper I, Berendsen HA, Bus SA. Optimising footwear for the diabetic foot: Data-driven custom-made footwear concepts and their effect on pressure relief to prevent diabetic foot ulceration. PLoS One 2020; 15(4): e0224010.

All the three references have been added in the reference list

Reviewer #4: Data-driven CAD-CAM vs traditional total contact custom insoles: a novel quantitative statistical
framework for the evaluation of insoles offloading performance in diabetic foot.


This study, in the field of the diabetic foot and neuropathic ulcer prevention, is important as it incorporates and develops the use of computer-aided design and manufacturing in the quantitative evaluation and design of custom-made orthotics for plantar pressure relief, in direct comparison to that for traditional total contact cast insoles.

The unique point of this study is the enhanced use of digital technology in this field, which is highly likely to be the way forward for any future standardised design of custom-made insoles for optimal pressure offloading.

We thank the reviewer #4 for her/his comments.

Major points:

Overall, sections could be shortened. Certain paragraphs would benefit from being made less wordy.

The paper has been intensively reworked following the reviewers’ suggestions and requests. We tried to shorten as much as possible the sections but unfortunately given the number of issues to be approached under the suggestions and requests of the reviewers it was not possible to fulfil such a suggestion truly.

Could the authors comment on their finding that the TCCI and CAD-CAM_CMI approaches did not produce good offloading in some cases (Fig. 5). What was unique about these individuals? Was it related to their specific characteristics such as foot deformities, gait etc? Or is this a methodology problem?

We added specific considerations in the Discussion section.

Please refer to lines 522-563

The methodology for obtaining the ‘blueprint’ images of the feet on a millimetric grid has not been detailed, however, I presume from Fig. 2 that the method involves manually drawing around the foot, then estimating the ‘areas of concern’ by observing an equivalent photo of the foot, then manually drawing the ROI’s onto the blueprint. This manual approach is not a standardised process and will undoubtedly introduce human error. The blueprint image and photo image in Fig. 2 are not even the same size for direct comparison. This is a study limitation and should be discussed. The authors may wish to draw comparisons to a recently published method which uses technology to standardise diabetic foot plantar images, developed to avoid such human error (eg. Yap et al, JDST, 2018).

We added specific considerations in the Discussion section.

Please refer to lines 495-503

The Results section is too long. Eight figures and 3 tables is very excessive. My initial thoughts are that Figs. 4 & 5 could be combined into one figure (a & b), as could Figs. 7 and 8. Data from tables 2 and 3 could be incorporated into the text.

Figure 7 and 8 have been removed and the text shortened. It has been impossible to combine the figures and the tables as suggested without compromising the readability of the paper and presented data.

There is no numerical data shown to back up the claims made about the statistical correlation of R-ROIs and at-risk locations (lines 383-387).

We added specific considerations in the Discussion section.

We claimed just that the correlation between at-risk areas marked on the blueprints and statistically determined R-ROIs was satisfying from a clinical point of view.

Please refer to lines 495-503

Minor points:

‘In-shoe dynamic planar pressure measurement’ (or something similar) may be a better sub-heading than ‘Instrumentation’.

Changed in “In-shoe dynamic plantar pressure measurement. “

There is repetition of methodology in lines 188-189 again at line 211-213.

Removed

Some sweeping statements in the Discussion should be re-phrased, to better indicate that this study’s results merely confirm or reflect results from other studies.
e.g. line 411 “This outcome is in perfect agreement with previous studies.”
line 416 “This result is in full agreement with the findings of other authors.”

The words: “perfect” and “full” were Removed
