# Supplementary material for: Data-driven CAD-CAM vs traditional total contact custom insoles: A novel quantitative-statistical framework for the evaluation of insoles offloading performance in diabetic foot
Source: PLoS One. 2021 Mar 4;16(3):e0247915. doi: 10.1371/journal.pone.0247915 (PMC7932531; doi:10.1371/journal.pone.0247915)
Supplement: Attachment — Submitted filename: Response to Reviewers PONE-D-20-14175_REVISION 2.docx [file pone.0247915.s003.docx]

PLOS ONE Decision: Revision required [PONE-D-20-14175R1] - [EMID:abb0860dbd965f93]

Posta in arrivo

PLOS ONE <em@editorialmanager.com>

27 ott 2020, 10:43

a me

CC: kevin.moerman@gmail.com, kevin.moerman@nuigalway.ie

PONE-D-20-14175R1

Data-driven CAD-CAM vs traditional total contact custom insoles: a novel quantitative-statistical framework for the evaluation of insoles offloading performance in diabetic foot

PLOS ONE

Dear Dr. D'Amico,

Thank you for submitting your manuscript to PLOS ONE. After careful consideration, we feel that it has merit but does not fully meet PLOS ONE’s publication criteria as it currently stands. Therefore, we invite you to submit a revised version of the manuscript that addresses the points raised during the review process.

Although reviewers 1 and 2 are happy to accept this submission, reviewers 3 and 4 have remaining concerns which have not been adequately addressed. Please accurately deal with their comments and appropriately respond to their queries in a clear and concise manor. One reviewer expresses concerns about the length of the article. Since other reviewers have, in this review process, asked for additional content it may be natural and acceptable that the article has grown in length. However I do urge you to consider this reviewer's concerns and aim at formulating sections in a more concise fashion where possible.

Please submit your revised manuscript by Dec 11 2020 11:59PM. If you will need more time than this to complete your revisions, please reply to this message or contact the journal office at plosone@plos.org. When you're ready to submit your revision, log on to https://www.editorialmanager.com/pone/ and select the 'Submissions Needing Revision' folder to locate your manuscript file.

Please include the following items when submitting your revised manuscript:

A rebuttal letter that responds to each point raised by the academic editor and reviewer(s). You should upload this letter as a separate file labeled 'Response to Reviewers'.

A marked-up copy of your manuscript that highlights changes made to the original version. You should upload this as a separate file labeled 'Revised Manuscript with Track Changes'.

An unmarked version of your revised paper without tracked changes. You should upload this as a separate file labeled 'Manuscript'.

If you would like to make changes to your financial disclosure, please include your updated statement in your cover letter. Guidelines for resubmitting your figure files are available below the reviewer comments at the end of this letter.

If applicable, we recommend that you deposit your laboratory protocols in protocols.io to enhance the reproducibility of your results. Protocols.io assigns your protocol its own identifier (DOI) so that it can be cited independently in the future. For instructions see: http://journals.plos.org/plosone/s/submission-guidelines#loc-laboratory-protocols

We look forward to receiving your revised manuscript.

Kind regards,

Kevin Mattheus Moerman, Ph.D.

Academic Editor

PLOS ONE

[Note: HTML markup is below. Please do not edit.]

Reviewers' comments:

Reviewer's Responses to Questions

Comments to the Author

1. If the authors have adequately addressed your comments raised in a previous round of review and you feel that this manuscript is now acceptable for publication, you may indicate that here to bypass the “Comments to the Author” section, enter your conflict of interest statement in the “Confidential to Editor” section, and submit your "Accept" recommendation.

Reviewer #1: All comments have been addressed

Reviewer #2: All comments have been addressed

Reviewer #3: (No Response)

Reviewer #4: (No Response)

2. Is the manuscript technically sound, and do the data support the conclusions?

The manuscript must describe a technically sound piece of scientific research with data that supports the conclusions. Experiments must have been conducted rigorously, with appropriate controls, replication, and sample sizes. The conclusions must be drawn appropriately based on the data presented.

Reviewer #1: Yes

Reviewer #2: Yes

Reviewer #3: Partly

Reviewer #4: Yes

3. Has the statistical analysis been performed appropriately and rigorously?

Reviewer #1: Yes

Reviewer #2: Yes

Reviewer #3: I Don't Know

Reviewer #4: Yes

4. Have the authors made all data underlying the findings in their manuscript fully available?

The PLOS Data policy requires authors to make all data underlying the findings described in their manuscript fully available without restriction, with rare exception (please refer to the Data Availability Statement in the manuscript PDF file). The data should be provided as part of the manuscript or its supporting information, or deposited to a public repository. For example, in addition to summary statistics, the data points behind means, medians and variance measures should be available. If there are restrictions on publicly sharing data—e.g. participant privacy or use of data from a third party—those must be specified.

Reviewer #1: Yes

Reviewer #2: Yes

Reviewer #3: Yes

Reviewer #4: Yes

5. Is the manuscript presented in an intelligible fashion and written in standard English?

PLOS ONE does not copyedit accepted manuscripts, so the language in submitted articles must be clear, correct, and unambiguous. Any typographical or grammatical errors should be corrected at revision, so please note any specific errors here.

Reviewer #1: Yes

Reviewer #2: Yes

Reviewer #3: No

Reviewer #4: Yes

6. Review Comments to the Author

Please use the space provided to explain your answers to the questions above. You may also include additional comments for the author, including concerns about dual publication, research ethics, or publication ethics. (Please upload your review as an attachment if it exceeds 20,000 characters)

Reviewer #1: thank you for your revisions. This is an excellent article that contributes to the offloading evidence for DFU.

Reviewer #2: (No Response)

Reviewer #3: Comments

The authors have done a large effort to revise the manuscript based on the reviewer’s comments, improving clarity. However, they do not always adequately respond to questions raised by the reviewers and often merely refer to the revised manuscript, where a response to the point raised is expected.

The authors introduce gait variability as argument to develop the QSF, but what is the clinical rationale for this? How does variability in pressure affect foot ulceration, suggesting that we need better methods that take into account these variabilities?

Line 284 – 286: “Such intrinsic variability connected to gait, both normal and pathological one, implies the necessity to approach the study of its characteristics from a statistical point of view. That is, by defining a rigorous averaging process to extract mean behaviours and their associated variability from which to derive clinically relevant parameters with a statistical significance.”

Ok, but to what extent do the authors fulfill this requirement?

Line 300-303: “We underline that such statistical R-ROIs determination permits to manage for the intrinsic gait variability considering both the mean value and its related SD. In such a way, the drawback of a simple direct threshold comparison performed on a single peak frame map is overcome by making the determination of R-ROIs position and extent a more theoretically rigorous process.”

The authors imply that the 200 kPa threshold method uses a single peak pressure frame map of the measurement of multiple step in-shoe pressure in the patient. This is not the case. A mean peak pressure map over a minimum 12 steps of in-shoe plantar pressure is used to identify regions with peak pressure >200 kPa and therefore also accounts for variability in pressure between steps, as reflected by the mean peak pressure over these steps. This should be corrected in the manuscript

What the 200 kPa threshold method does not do is use the SD of the mean peak pressure, but from the paper it is not clear yet to me in what exact way the QSF does take the SD into account and why that is important beyond from a theoretical point of view. How does this work exactly?

ANSWER

***************************************************************************************

We thank the reviewer for the above group of questions posed. Being them basically all connected to each other we think is better to give a comprehensive answer for all of them.

We think that the clinical rationale for the introduction of such complex QSF is aimed to have the most conservative as possible approach in considering any possible area of risk. This is accomplished only if gait variability is considered in its complete expression.

It is true, as the reviewer points out, that variability information is partially considered in the computed mean peak pressure map. But processing this information through a simple direct comparison to the 200kPa threshold could lead to neglect some of the possible risk areas, as the standard deviation information is not considered, for its statistical contribution, as is the case in the QSF approach.

To better explain such a concept, we included a simple numerical example. So, we modified/added to the manuscript the following: (lines 282-290 of the marked manuscript)

*“In such a way, the drawback of a simple direct threshold comparison performed on a mean peak frame map is overcome by making the determination of R-ROIs position and extent a theoretically more rigorous process. Indeed, a series of cells having average values fairly below the 200 kPa threshold but with high SD (let us say µ=170kPa±30kPa where SD=30kPa) would definitely not be considered by the direct threshold method. Conversely, if the variability of gait were found to be high producing a high SD (as in the numerical example), the one-sample t-test (included in the QSF) would likely consider this series of cells not statistically different from the 200 kPa threshold. Thus, the potential risk associated with this series of cells is not neglected by including them in the R-ROIs. For these reasons, the QSF approach is more conservative considering the statistical risk.”*

We also added in the Discussion section (lines 486-490 of the marked manuscript) the following considerations about the possible sources of variability that can affect the underfoot pressure:

*“It is useful to emphasize that there are two possible sources of variability: the first and most intuitive is connected to the poor repeatability of each gait cycle in terms of both speed and length of the steps. The second relates to the continuous variation of the foot support modality, which is modulated, step by step, according to the balance variations and which can be particularly poorly controlled when peripheral neuropathy is present.”*

In addition, about the number of steps to be used to calculate the mean peak pressure map, the work of Arts et al. 2011 [41] suggest a minimum of 12 steps. However, “a mean peak pressure map over a minimum 12 steps of in-shoe plantar pressure used to identify regions with peak pressure >200 kPa” provides inadequate statistical power when the described QSF is used. We described already such situation also from a numerical point of view in the Discussion section (lines 503-512 of the marked manuscript) that we report also here for clarity:

*“However, some requirements must be fulfilled. In particular, using a prior power calculation analysis, we determined that at least 24 mid steps per each foot must be recorded. Indeed, such a sample size is necessary to discriminate differences of about 0.5 SD between means (i.e. the t-Test on each pressure cell for custom-made vs flat insole comparisons - Cohen’s d=0.6) or respect to a fixed threshold (i.e. the mean peak pressure cell vs the 200 kPa threshold through One-Sample t-Test - Cohen’s d=0.52) keeping the test power at 80%, and the type I error at α=5% levels respectively. Such a requirement doubles the 12 steps limit suggested by the work of Arts et al. 2011 [41] that, if applied to our QSF, would significantly reduce the power of the test (at equal effect size). I.e. down to 47% for the t-Test comparison and 51% for the 200 kPa threshold through One-Sample t-Test comparison [42–44]”*

***************************************************************************************

The design process of the CADCAM insoles is quite similar to Owing et al.(ref 9), but there is no reference to their approach in the methods section. This should be added

ANSWER

***************************************************************************************

We thank the reviewer for such a note. We added the following sentences at lines 199-202 of the marked manuscript:

*“Such a method is similar to that presented by Owings et al. [9]. The main substantial differences, in the actual CAD-CAM_CMI design approach, are related to the use of baropodometric insoles to determine the mean pressure maps driving the insole design and to the statistically rigorously determined number of necessary steps to be averaged (see below).”*

***************************************************************************************

Line 307-308. “For such a reason we did not give any a priori particular importance to specific anatomical regions.” Why not? Since specific regions are much more susceptible for ulceration than other. For example, in-shoe pressures in the heel are often among the highest found in the foot, but ulcers hardly ever occur on the plantar heel, so less important for the analysis and outcome

ANSWER

***************************************************************************************

We thank the reviewer for the questions. We clarified the concept lengthening the explanation in the Data Analysis section (lines 294-300 of the marked manuscript):

*“For such a reason we did not give any a priori particular importance to specific anatomical regions. Conversely, we left that the statistical determination of the size and position of the R-ROIs would indicate which of the anatomical structures would assume a particular criticality for each patient. So it was not necessary to pre-process pressure data applying a mask to identify any given foot region anatomical structure [9,40,41]. In such a way, all the possible risk areas are taken into account, letting the clinician to evaluate them all and to identify each possible critical issue.”*

Furthermore, the independence of the QSF from the predetermined specific anatomical regions pre-processing reveals to be useful to analyse the underfoot pressure redistribution induced by the custom-made foot orthoses as remarked in the Discussion section (lines 640-648 of the marked manuscript):

*“The proposed technique presents another positive feature, as it allows the possibility to determine on a statistical base the underfoot pressure redistribution induced by the custom-made foot orthosis. The evaluation of such redistribution allows the assessment of the manufactured foot orthoses performance highlighting new eventual R-ROIs to be furtherly removed. Such a process could be iterated up to the complete removal of R-ROIs. In such a way the QSF approach could demonstrate useful to help preventing the risk of ulceration. Furthermore, our technique allows the analysis of the whole plantar surface area of the foot, with the single pressure cell detail, independently from a predetermined anatomical identification/masking.”*

***************************************************************************************

Based on their outline of comparisons between the QSF and the >200 kPa threshold method, their statement that “From all the above considerations, the direct 200kPa threshold appears theoretically

inadequate to grant the appropriate at-risk regions identification.” is too bold. See above comments.

ANSWER

***************************************************************************************

We thank the reviewer for such a note. We modified the text as the following (lines 512-515 of the marked manuscript):

*“From all the above considerations, the QSF approach takes into account peak pressure variability during gait so granting from a statistical point of view a well controlled level of test power and the type I error in the identification of the risk regions. Such a framework is more rigorous than performing a direct 200kPa threshold on the average of peak pressure maps.”*

***************************************************************************************

Furthermore, The 200kPa level method has been proven very useful in optimizing diabetic footwear and has also been identified as an appropriate threshold below which foot ulcers can be prevented when the footwear is worn (ref 41 in their paper). The QSF has not yet shown clinical relevance, merely a biomechanical or theoretical one, and since the aim of custom-made footwear is to reduce risk of ulceration, the authors should be careful with stating that demonstrated clinically relevant methods are inadequate. At most, the author may present the QSF as an alternative method that takes variability in peak pressure more into account in a statistical way and that it remains to be proven what the clinical relevance of this method is in reducing risk of ulceration

ANSWER

***************************************************************************************

We thank the reviewer for such a note. We modified the text in the Discussion section as the following (lines 606-608 of the marked manuscript):

*“For this reason, extended clinical trials have to be planned to establish the clinical relevance of the QSF method is in reducing risk of ulceration, and validate the proposed methodology as a routine clinical tool, on a larger scale”.*

***************************************************************************************

Conclusions:

Line 703: “Indeed, the method demonstrated its usefulness in identifying high-risk areas with ascertained statistical significance (R-ROIs) improving the direct 200kPa cut-off approach outlined in the literature.”

Should be modified based on above argumentation

ANSWER

***************************************************************************************

We thank the reviewer for such a note. We modified the text in the Conclusions section as the following (lines 637-640 of the marked manuscript):

*“Indeed, the QSF method demonstrated its usefulness in identifying high-risk areas by taking into account peak pressure variability during gait with ascertained statistical significance, a controlled level of test power and type I error in more rigorous way than performing a direct 200kPa threshold on the average of peak pressure maps.”*

***************************************************************************************

Line 705: “The proposed technique presents another positive feature, as it allows the possibility to

determine on a statistical base the underfoot pressure redistribution induced by the custom-made

foot orthosis, thus preventing the risk of ulceration in different underfoot regions.”

This reads as if the technique itself reduces the risk of ulceration, where the authors mean to say I think that with the technique better offloading insoles can be designed that may reduce risk of ulceration. This should be better reflected in the conclusions

ANSWER

***************************************************************************************

We thank the reviewer for such a note. We modified the text in the Conclusions section as the following (lines 640-645 of the marked manuscript):

*“The proposed technique presents another positive feature, as it allows the possibility to determine on a statistical base the underfoot pressure redistribution induced by the custom-made foot orthosis. The evaluation of such redistribution allows the assessment of the manufactured foot orthoses performance highlighting new eventual R-ROIs to be furtherly removed. Such a process could be iterated up to the complete removal of R-ROIs. In such a way the QSF approach could demonstrate useful to help preventing the risk of ulceration.”*

***************************************************************************************

Authors should check their references: some papers seem to appear twice in the reference list

ANSWER

***************************************************************************************

We thank the reviewer for such a note. We removed duplications from the References.

***************************************************************************************

Authors should also check grammar and typo's and English writing in the paper

ANSWER

***************************************************************************************

We thank the reviewer for such a note. We reprocessed all the manuscript in multiple ways also using specific software for language check.

***************************************************************************************

Reviewer #4: I requested that manuscript sections could be shortened. However, I find that many of the new phrases added to the various sections are over-elaborate, sometimes a little confusing to read, and have added considerably to the word count.

ANSWER

***************************************************************************************

We thank the reviewer for such a note. Indeed, we agree and we tried to shrink the text as much as possible, but to answer to all the questions posed by the reviewers made to shorten the text a real hard task.

***************************************************************************************

I asked a specific question regarding why the TCCI and CAD-CAM-CMI approaches did not

produce good offloading in some cases, and speculated if it was related to individuals’ specific characteristics such as foot deformities, gait etc? Or a methodology problem? The authors referred me to a new, 40-line paragraph in the Discussion; however, this does not appear to answer my specific question.

ANSWER

***************************************************************************************

We thank the reviewer for such a note. We are sorry in effect we answered to the question, it is a complex question that needs to be answered at length with various considerations. Unluckily the answer was included in the manuscript at different lines we previously indicated. We are sorry for the error.

The answer is contained in the Discussion section at lines 549-566 of actual marked manuscript. We report here the extract to avoid any further error.

*“Indeed, although CAD-CAM_CMI produced a better outcome in R-ROIs removal/reduction respect to TCCI, still it yielded 39 sub-optimal results out of 60 feet considered (65%). Multiple causes can be argued to explain such a sub-optimal outcome. First, as a general consideration, the information obtained through pressure and 3D shape measuring process is fundamental, but it is not sufficient to completely describe the complexity of the phenomenon under study. Indeed, a recent study proved that using additional information, data-driven modifications improve the offloading efficacy on handmade and CAD-CAM design for patients with diabetic neuropathy at high risk of foot ulceration [25]. In particular, the study showed that the best results could be achieved even in a handmade approach if a well-structured procedure is applied. Such a procedure consisted in a detailed algorithm based on the knowledge that specifies the design elements and materials used, their hardness, thickness, and location and in-shoe plantar pressure guided insole/footwear modifications (multiple subsequent modifications are applied when pressure targets are not achieved) to further improve the insole/footwear after delivery [25]. The offloading algorithm used for the CAD-CAM insole design in this study is based on some proportional deepening under the R-ROIs areas. Such an approach could be undoubtedly improved by including computations that integrate material dynamic response characteristics. This latter, combined with novel patient-specific finite element modelling of the human foot into the algorithm [24,47] could reasonably help to advance CAD-CAM insole design. This is a matter for further study.*

***************************************************************************************

My other queries have been answered satisfactorily.

7. PLOS authors have the option to publish the peer review history of their article (what does this mean?). If published, this will include your full peer review and any attached files.

If you choose “no”, your identity will remain anonymous but your review may still be made public.

Do you want your identity to be public for this peer review? For information about this choice, including consent withdrawal, please see our Privacy Policy.

Reviewer #1: Yes: Richard Collings

Reviewer #2: No

Reviewer #3: No

Reviewer #4: No

[NOTE: If reviewer comments were submitted as an attachment file, they will be attached to this email and accessible via the submission site. Please log into your account, locate the manuscript record, and check for the action link "View Attachments". If this link does not appear, there are no attachment files.]

While revising your submission, please upload your figure files to the Preflight Analysis and Conversion Engine (PACE) digital diagnostic tool, https://pacev2.apexcovantage.com/. PACE helps ensure that figures meet PLOS requirements. To use PACE, you must first register as a user. Registration is free. Then, login and navigate to the UPLOAD tab, where you will find detailed instructions on how to use the tool. If you encounter any issues or have any questions when using PACE, please email PLOS at figures@plos.org. Please note that Supporting Information files do not need this step.
